# Organizational culture, trust, and supervisor support: drivers of employee commitment in China

**DOI:** 10.3389/fpsyg.2025.1548514

**Published:** 2025-11-27

**Authors:** Xiaoyan Zhang, Youpeng Gao

**Affiliations:** 1 School of Humanities, Shanghai Jiao Tong University, Shanghai, China; 2 School of Media & Communication, Shanghai Jiao Tong University, Shanghai, China

**Keywords:** organizational culture, supervisor support, organizational trust, affective commitment, employee commitment, workplace dynamics

## Abstract

**Introduction:**

This study addresses a critical gap by investigating the complex interplay between organizational culture, supervisor support, and organizational trust in fostering affective commitment among employees, particularly within the distinct cultural context of mainland China.

**Methods:**

Data were collected from 439 participants through an online survey instrument. Hierarchical regression analysis was used to examine the direct and interactive effects of the independent variables on affective commitment.

**Results:**

Hierarchical regression analysis revealed that clan and hierarchy cultures, along with supervisor support and organizational trust, were significant positive predictors of affective commitment. Adhocracy and market cultures did not show significant direct effects. Notably, two significant interaction effects emerged: one between supervisor support and organizational trust, and another between clan culture and organizational trust. Simple slopes analysis indicated that high levels of supervisor support significantly strengthen the positive association between organizational trust and affective commitment, and that a strong clan culture similarly strengthens this relationship.

**Discussion:**

These findings underscore the importance of fostering a supportive supervisory environment and building trust within organizations, particularly when supported by collectivist and structured cultural attributes, to enhance employee commitment. Theoretical and practical implications are discussed, highlighting the need for cultivating clan and hierarchy-oriented cultures, strengthening organizational trust, and implementing effective supervisor training programs.

## 1 Introduction

Affective commitment, characterized by an employee’s emotional attachment and identification with their organization, is paramount for organizational success (Mercurio, 2015; Meyer and Allen, 1991). This strong bond drives critical positive outcomes, including enhanced job performance, reduced turnover, and increased organizational citizenship behaviors (Meyer et al., 2002; Meyer and Herscovitch, 2001). In the current volatile global environment, marked by unprecedented talent mobility and constant disruption, fostering significant emotional investment is no longer merely advantageous but essential for organizational resilience, agility, and innovation (Klein et al., 2021).

Extensive research identifies organizational culture, supervisor support, and organizational trust as key antecedents of affective commitment (Top et al., 2013; Vanhala et al., 2016). Organizational culture, operationally defined here by shared assumptions, values, and practices that guide organizational members (Cameron and Quinn, 2006), consistently links to commitment, particularly cultures emphasizing teamwork and support (Aranki et al., 2019; Carvalho et al., 2018; Dwivedi et al., 2014). Supervisor support, operationally defined as employees’ perceptions that their supervisors value their contributions, care for their well-being, and offer necessary assistance (Frear et al., 2018), significantly predicts commitment and enhances retention (Alkhateri et al., 2018; Kalidass and Bahron, 2015; Wang, 2014). Organizational trust, operationally defined as employees’ belief that their organization will act in their best interests and will not intentionally harm them (Nyhan and Marlowe, 1997), also plays a pivotal role, fostering emotional attachment through job satisfaction and fair practices (Malla and Malla, 2023; Top et al., 2013; Vanhala et al., 2016).

Despite the well-established individual contributions of these factors, a critical theoretical gap persists in comprehending their interdependent interplay in shaping affective commitment. Traditional scholarship often adopts an additive perspective, overlooking how the combined and conditional effects of organizational culture, supervisor support, and organizational trust may synergistically amplify or attenuate their influence. Specifically, while existing studies often treat these constructs as isolated predictors, there is a lack of understanding regarding the mechanisms through which a supervisor’s direct support might modify the broader impact of an organization’s inherent culture or its prevailing level of trust on employee commitment. This study directly addresses this void by rigorously investigating the moderating role of supervisor support, theorizing it acts as a crucial contingency factor that amplifies the impact of organizational trust and organizational culture on affective commitment. This perspective suggests that the direct interactions employees have with their supervisors can significantly shape how broader organizational characteristics are perceived and internalized, thereby influencing their emotional attachment.

Furthermore, situating this research within mainland China offers unique theoretical and practical insights. The predominant body of commitment literature is rooted in Western, individualistic cultural frameworks. However, China’s distinctive collectivist values, pronounced hierarchical structures, and the pervasive influence of guanxi (interpersonal connections) (Hofstede, 2001; Jiang et al., 2017) suggest that the mechanisms through which organizational culture, supervisor support, and organizational trust foster commitment may operate substantially differently. Understanding these context-specific dynamics is not just an academic exercise; it is crucial for developing effective talent management strategies in China’s rapidly evolving and economically significant workforce. Our study’s rationale, therefore, is to contribute to a more globally representative organizational theory by examining these relationships in a significant non-Western cultural context, thereby enhancing the practical applicability of commitment research.

To achieve these aims, this quantitative study employs a survey methodology, collecting data from 439 employees across diverse industries in mainland China. Utilizing hierarchical regression analysis, we examine the independent and interactive effects of organizational culture, supervisor support, and organizational trust on affective commitment. This rigorous approach offers a nuanced exploration of the complex mechanisms driving employee loyalty and engagement, providing evidence-based guidance for culturally attuned human resource practices and contributing to a more comprehensive, globally informed theory of organizational behavior.

The remainder of this paper is structured as follows. Section 2 reviews the relevant literature and develops the theoretical framework, culminating in the specific hypotheses and research questions. Section 3 details the research methodology, including participant characteristics, instruments, and data analysis procedures. Section 4 presents the descriptive statistics, correlations, and the results of the hierarchical regression analysis. Finally, Section 5 discusses the theoretical and practical implications of our findings, acknowledges the study’s limitations, and proposes directions for future research.

## 2 Literature review

### 2.1 Affective commitment

Affective commitment, defined as an employee’s emotional attachment and identification with their organization, stands as a critical pillar of organizational success (Mercurio, 2015; Meyer and Allen, 1991). This emotional bond compels employees to remain engaged by genuine desire, rather than mere obligation, fostering behaviors that directly align with long-term organizational objectives (Allen and Meyer, 1996; Mercurio, 2015). This intrinsic motivation and alignment enable employees to surpass basic expectations and actively contribute to organizational success (Bandura, 1988; Matzler and Renzl, 2007). Distinct from continuance commitment (driven by perceived costs of leaving) and normative commitment (rooted in moral obligation), affective commitment is uniquely propelled by intrinsic motivation and a deep alignment between individual values and organizational goals (Brown et al., 2019; Chang et al., 2012). This characteristic highlights its singular importance in cultivating notable emotional investment and sustained engagement within the workplace.

A comprehensive understanding of affective commitment necessitates a focused examination of its core antecedents. While various factors contribute to this vital emotional bond (Meyer et al., 2002), our study specifically investigates three highly interdependent constructs that are fundamental to an employee’s organizational experience: organizational culture, supervisor support, and organizational trust. These elements represent critical levers for cultivating the psychological states necessary for strong affective commitment. While much of this foundational work establishes the direct links between these factors and commitment, a more nuanced understanding of their interplay, especially within diverse cultural contexts, remains an active area of inquiry.

### 2.2 Organizational culture and its role in commitment and performance

Organizational culture, defined by shared assumptions, significantly influences employee commitment and adaptability (Marker, 2009; Schein, 2010). Although foundational scholarship (e.g., Cook and Wall, 1980; Denison, 1990) primarily emphasized culture’s role in promoting internal consistency and coordination, contemporary research increasingly highlights the critical importance of adaptability and flexibility. This shift reflects an evolving understanding, stressing how organizations must remain resilient while preserving core values amidst dynamic environments (Anning-Dorson, 2021; Collins et al., 2005; Gochhayat et al., 2017; Marker, 2009; Orishede and Igbigbisie, 2022). This evolution points to cultures that adeptly balance stability with dynamic response.

Theoretical perspectives clarify how culture fosters commitment by aligning with organizational goals. Schein’s (2010) framework, for instance, explores how robust cultures can evolve while maintaining coherence. Denison’s (1990) hypotheses on consistency, mission, involvement, and adaptability further explain how shared values and adaptive norms contribute to organizational resilience. These frameworks underscore culture’s role in guiding employee behaviors and shaping their psychological attachment.

Organizational culture directly influences employee affective commitment through value congruence. Employees perceiving alignment between their personal values and organizational norms experience greater emotional attachment (Aranki et al., 2019; Kawiana et al., 2018). Inclusive cultures, particularly when supported by transformational leadership, are notably effective in deepening emotional bonds and strengthening commitment (Ashikali and Groeneveld, 2015), fostering a sense of belonging vital for sustained affective commitment.

The impact of specific cultural dimensions on commitment is nuanced and context-dependent. For example, in public service, cultures emphasizing societal impact enhance commitment among intrinsically motivated employees (Austen and Zacny, 2015). Globally, core values like shared purpose consistently predict loyalty (Chordiya et al., 2017). In dynamic industries, an entrepreneurial orientation fosters engagement and commitment through innovation (Soomro and Shah, 2019). These findings underscore that different cultural dimensions uniquely contribute to affective commitment. While many studies broadly link culture to various outcomes like job performance (Zeb, 2020), understanding the specific cultural typologies most relevant to *affective commitment* in non-Western contexts remains crucial. For instance, Zeb et al. (2022) explored how competing value framework cultures (adhocracy, market, clan, hierarchy) influence job performance, finding varying effects and mediating roles for HR practices. Extending this line of inquiry to commitment, considering the Chinese context, where collectivist values, stability, and harmony are deeply ingrained, identifying which cultural orientations specifically foster affective commitment is crucial. This empirical and theoretical imperative leads to our hypothesis regarding the association between specific organizational culture dimensions and affective commitment.

*H1*: Specific organizational culture dimensions are positively associated with employee affective commitment.

### 2.3 Organizational trust

Organizational trust, broadly defined as employees’ belief that their organization will act in their best interests and will not intentionally harm them, is a foundational element in workplace dynamics (Blomqvist, 1997; Gilbert and Tang, 1998; Parra et al., 2011). This trust develops through a social exchange process where employees evaluate management based on their experiences (Blau, 2017; Jeong and Oh, 2017), and is shaped by systemic factors like job security and fair evaluations (Cho and Park, 2011). High trust levels are associated with increased productivity (Nyhan and Marlowe, 1997), while low trust can lead to inefficiencies (Singh and Srivastava, 2016) and stress (Hurley, 2012).

Trust in organizations can be directed towards individuals (e.g., supervisors, peers) or the organization as a whole (Bachmann and Inkpen, 2011; Gustafsson et al., 2021). Interpersonal trust, whether vertical (in managers) or lateral (among colleagues), depends on perceived competence, benevolence, or reliability (Tan and Lim, 2009; Vanhala et al., 2016). Specifically, organizational trust encompasses confidence in leadership, organizational objectives, and the belief that company actions benefit the workforce (Gustafsson et al., 2021; Nedkovski et al., 2017). This perception reduces ambiguity and uncertainty within a trusting culture (Kannan-Narasimhan and Lawrence, 2012; Taylor et al., 2023).

Organizational trust is consistently linked to positive workplace outcomes, particularly affective commitment (Bastug et al., 2016; Frazier et al., 2015; Malla and Malla, 2023; Mayer et al., 1995; Ugwu et al., 2014; Vanhala et al., 2016). An emotional bond with the organization, characteristic of affective commitment, is significantly fostered by high levels of trust, leading to stronger employee loyalty and reduced turnover (Bastug et al., 2016; Vanhala et al., 2016). Trust in leadership, specifically, is strongly linked to affective commitment, and organizational identification is deepened when employees internalize values in a trusting environment (Ng, 2015). Peer trust also significantly predicts affective organizational commitment (Ferres et al., 2004). Meta-analytic research further highlights the influence of interpersonal trust on organizational commitment, especially during periods of instability (Dirks and Ferrin, 2001). Based on this evidence, we hypothesize that a higher level of organizational trust will directly lead to stronger affective commitment among employees.

*H2:* Organizational trust is positively associated with employee affective commitment.

Cross-cultural studies reveal that trust’s influence on commitment is culturally nuanced. For instance, in collectivist cultures like China and South Korea, trust is often tied to perceptions of fairness, a contrast to individualistic cultures where leadership behaviors might be more central (Jiang et al., 2017). This indicates the crucial role of cultural context in shaping the trust-commitment relationship. In high-stress sectors such as healthcare, trust, particularly in leadership and systems, mediates the relationship between organizational justice and employee commitment, proving vital for staff morale and resilience (Chen et al., 2015). These cross-cultural variations underscore the need to study trust dynamics in specific national contexts, a point reinforced by research examining the influence of organizational justice on job performance in developing contexts, often through mediating HR practices (Zeb et al., 2021). While their focus differs, such studies highlight the complex interplay of factors within culturally distinct organizational environments. This evidence, along with the close theoretical ties between organizational culture and trust, leads us to pose a research question to examine their interaction in the Chinese context.

*RQ1*: How does the interaction between organizational culture and organizational trust influence employee affective commitment?

### 2.4 Supervisor support and its role in fostering affective commitment

Supervisor support, defined as employees’ perception that their supervisors value their contributions, care for their well-being, and offer necessary assistance, significantly shapes various work outcomes (Frear et al., 2018; Maertz et al., 2007). Research consistently shows that supportive supervisors directly enhance affective commitment, alongside job satisfaction and organizational loyalty, thereby reducing turnover and improving performance. This strong link often stems from supervisors’ ability to address subordinates’ emotional needs, which is critical for fostering organizational commitment (Dawley et al., 2008; Ng and Sorensen, 2008). Emotionally intelligent supervisors cultivate environments conducive to stronger employee commitment. Recent scholarship has further underscored the importance of supervisor support in driving positive employee outcomes, including job performance, often through psychological mechanisms like self-efficacy and empowerment (Zeb et al., 2024; Zeb et al., 2025).

Employees often perceive supervisors as direct extensions of the organization, interpreting their behavior as reflective of company values and intentions (Tannenbaum, 2013). This perspective aligns with Organizational Support Theory (OST), which posits that supervisors’ actions directly influence employees’ perceptions of overall organizational support (Casimir et al., 2014; Eisenberger et al., 2002; Rothmann and Cooper, 2015). Consequently, the direct link between supervisor support and affective commitment extends beyond mere interpersonal dynamics to reflect perceived organizational backing. When employees perceive their supervisors as genuinely caring, this sentiment often generalizes to the organization itself, strengthening emotional attachment (Eisenberger et al., 2020; Rhoades and Eisenberger, 2002). Based on this, we hypothesize a direct relationship:

*H3:* Perceived supervisor support is positively associated with employee affective commitment.

Beyond its direct impact, supervisor support plays a multifaceted role in reinforcing other commitment antecedents. High levels of supervisor support correlate with reduced burnout (Weigl et al., 2016) and are strong predictors of job satisfaction (Munn et al., 1996), which, in turn, mediates its relationship with reduced turnover intentions (Alkhateri et al., 2018). The bidirectional dynamic with perceived organizational support (POS) further suggests that multi-level support amplifies positive effects on commitment (Maertz et al., 2007; Yoon and Thye, 2002). In various sectors, supervisor support enhances affective commitment, reducing turnover (Nichols et al., 2016; Orgambídez and Almeida, 2020). Crucially for this study, supervisor support has also been shown to moderate the relationship between organizational justice and affective commitment, amplifying the positive impact of fairness in contexts like Chinese higher education (Li, 2020; Li et al., 2018). This highlights how supervisors create psychologically safe and trusting environments (Gao et al., 2022) that deepen emotional attachment, suggesting its potential to interact with both organizational culture and organizational trust. Thus, while supervisor support’s direct role is well-established, understanding its conditional effects when interacting with broader organizational characteristics like culture and trust is critical for a comprehensive model of employee commitment. We therefore propose the following interactive hypotheses:

*H4*: Perceived supervisor support moderates the relationship between specific organizational culture dimensions and employee affective commitment, such that the positive association between favorable cultural attributes (e.g., clan, hierarchy) and affective commitment is significantly stronger under conditions of high perceived supervisor support.

*H5*: Perceived supervisor support moderates the relationship between organizational trust and employee affective commitment, such that the positive association between organizational trust and affective commitment is substantially stronger under conditions of high perceived supervisor support.

### 2.5 Theoretical framework

The foundational importance of affective commitment is well-established in the literature. While extant research identifies organizational culture, supervisor support, and organizational trust as key antecedents, traditional approaches often treat these factors in isolation. Our study addresses a critical theoretical gap by examining their conditional and interdependent effects. Specifically, we propose that perceived supervisor support acts as a pivotal contingency factor, modulating the influence of both the broader organizational culture and the pervasive organizational trust climate on employee commitment. Supervisors are not merely sources of direct personal support; they also serve as critical interpretive agents, translating organizational policies and trustworthiness into tangible daily experiences. Their supportive behaviors can therefore either amplify or attenuate the positive effects derived from macro-level organizational characteristics.

This investigation is further grounded within mainland China’s distinctive cultural landscape, which provides a unique context to test these relationships. China’s deeply ingrained collectivist values, respect for hierarchical structures, and the intricate web of guanxi strongly suggest that the mechanisms through which culture, support, and trust foster commitment may operate differently than in Western, individualistic contexts. By empirically examining these relationships within the Chinese milieu, our study aims to challenge universalistic assumptions and contribute to a more globally representative understanding of organizational behavior.

**Figure 1:**
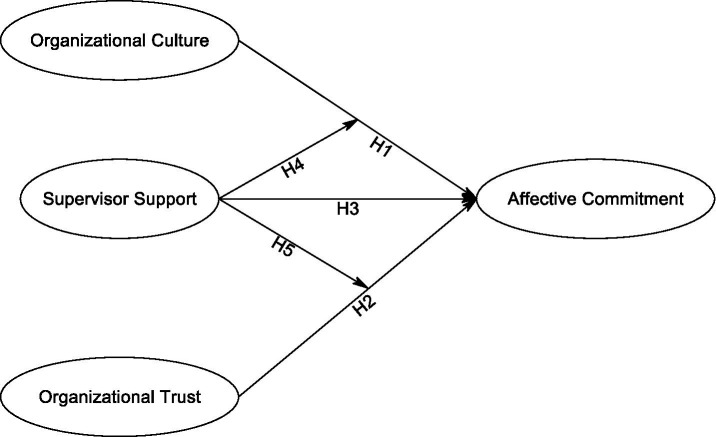
Conceptual model of affective commitment antecedents and their interactions.

Based on this integrated framework, we articulate a conceptual model (Figure 1) that visually represents the hypothesized direct relationships and the critical interactive effects under investigation.

## 3 Methods

### 3.1 Participants

A purposive sampling approach was employed to target a representative sample of the Chinese workforce. Inclusion criteria specified that participants be at least 18 years old, hold a full-time position within their current organization for a minimum of 6 months, and possess fluency in Mandarin Chinese, the survey language. From the initial pool of potential participants, a total of 439 individuals completed the survey, yielding a usable sample size (*n* = 439) that reflected the national workforce demographics. This diverse group included 41% male (*n* = 180) and 59% female (*n* = 259) participants, with an average age of 32.4 years old (SD = 7.8). The age range spanned from 18 to 58 years, falling squarely within the typical working age range in mainland China. Furthermore, participants possessed an average of 5.2 years of experience (SD = 3.1) within their current organizations, suggesting a level of familiarity with their organizational culture and supervisor dynamics. The sample also encompassed a diverse spectrum of industries across China, including technology (22%), manufacturing (18%), finance (15%), healthcare (12%), education (10%), and other service industries (23%). This distribution reflects the country’s growing service sector while acknowledging the continued presence of established industries.

### 3.2 Instruments

To ensure linguistic and cultural equivalence, all instruments were translated from English to Mandarin using a standard back-translation procedure (Brislin, 1970). Initially, the scales were translated into Mandarin by a bilingual expert. A second independent translator, unfamiliar with the original English versions, then back-translated the instruments into English. Any discrepancies between the original and back-translated versions were discussed and resolved through consensus to ensure conceptual alignment and cultural relevance of the items. Furthermore, to verify the construct validity of the translated scales, Confirmatory Factor Analyses (CFAs) were conducted for each instrument.

#### 3.2.1 Organizational trust scale

Organizational trust was assessed using the 12-item Organizational Trust Inventory (OTI; Nyhan and Marlowe, 1997), which includes two dimensions: trust in the supervisor (8 items) and trust in the organization (4 items). Example items include: “My level of confidence that my supervisor will make well thought out decisions about his or her job is high” for trust in the supervisor, and “The level of trust between supervisors and workers in this organization is high” for trust in the organization. Reliability coefficients were strong, with Cronbach’s alpha values of 0.97 for trust in the supervisor and 0.87 for trust in the organization. CFA results indicated a good fit for the Organizational Trust Scale: χ^2^/df = 2.31, CFI = 0.95, TLI = 0.93, RMSEA = 0.06 (90% CI = 0.05–0.08), SRMR = 0.04. For the purpose of our main analyses, these two dimensions were combined to form a single, overarching “Organizational Trust” construct (*α* = 0.94), reflecting employees’ general perception of trustworthiness within their workplace environment. This aggregation is consistent with previous research that uses a composite trust score when examining its broader influence on organizational outcomes.

#### 3.2.2 Affective commitment scale

Affective commitment was measured using six items from Allen and Meyer’s (1996) scale. Sample items include: “I would be thrilled to spend the rest of my career with this organization” and “I feel as if this organization’s problems are my own.” The reliability of the scale was high, with a Cronbach’s alpha of 0.91. CFA results demonstrated good fit: χ^2^/df = 2.20, CFI = 0.96, TLI = 0.94, RMSEA = 0.05 (90% CI = 0.04–0.07), SRMR = 0.03.

#### 3.2.3 Organizational culture assessment instrument

Organizational culture was assessed using the Organizational Culture Assessment Instrument (OCAI) developed by Cameron and Quinn (2006), which measures four distinct cultural dimensions, each comprising six items. The clan dimension (α = 0.82) captures the extent to which the organization emphasizes teamwork, consensus, and participation, as exemplified by statements such as “The management style in the organization is characterized by teamwork, consensus, and participation.” The adhocracy dimension (α = 0.78) reflects an entrepreneurial and dynamic environment, with items like “The organization is a very dynamic and entrepreneurial place. People are willing to take risks.” The market dimension (α = 0.81) focuses on results-oriented behavior, as demonstrated by the item “The organization is very results-oriented. A major concern is with getting the job done.” Finally, the hierarchy dimension (α = 0.79) measures stability and conformity within the organization, with statements such as “The management style in the organization is characterized by security of employment, conformity, and stability in relationships.” CFA results confirmed the construct validity of the OCAI, yielding fit indices that indicated a good model fit: χ^2^/df = 2.45, CFI = 0.94, TLI = 0.92, RMSEA = 0.06 (90% CI = 0.05–0.08), and SRMR = 0.04. These results validate the reliability and robustness of the instrument in assessing organizational culture within the context of this study.

#### 3.2.4 Perceived supervisor support scale

Perceived supervisor support was assessed using the four-item scale adapted from Rhoades et al. (2001). An example item is: “My supervisor cares about my well-being.” The scale has been validated in Chinese samples and showed strong reliability in this study (Cronbach’s α = 0.92). The CFA results indicated a good model fit: χ^2^/df = 2.12, CFI = 0.96, TLI = 0.94, RMSEA = 0.05 (90% CI = 0.03–0.07), SRMR = 0.03.

### 3.3 Procedure

The study adhered to ethical guidelines established by Shanghai Jiao Tong University, with Institutional Review Board (IRB) approval secured prior to data collection. Data were collected from September to December 2023 using Qualtrics, a secure online platform that ensured participant privacy and anonymity.

Before launching the main survey, a pilot test was conducted with a diverse group of 30 participants (*n* = 30) to refine the survey instrument and ensure its functionality and clarity. Feedback from this pilot group led to minor wording adjustments and the addition of a Likert scale tutorial, ensuring a smooth and comprehensible survey experience for subsequent participants.

Following the pilot test, the main data collection was initiated. This involved collaborating with human resource departments across various industries in mainland China to distribute recruitment emails to their full-time employees. The email clearly outlined the study’s objectives, emphasized the voluntary nature of participation, and guaranteed confidentiality and anonymity of all responses. Electronic informed consent was obtained from all participants before they proceeded with the online survey. A small, IRB-approved incentive (e.g., raffle entry) was offered to enhance response rates. Qualtrics features, including mandatory fields and logic branching, were utilized to ensure data quality and prevent duplicate responses. Participation was monitored, with additional outreach to HR departments initiated as needed to improve response rates.

### 3.4 Data analysis

Survey responses were imported into SPSS for analysis, and data cleaning procedures were applied to address missing values and outliers. Missing data accounted for less than 2% of the total, and listwise deletion was used to ensure that subsequent analyses were based on complete cases. Outliers were identified using boxplot visualizations and standard deviation analysis, with cases exceeding 3.5 standard deviations from the mean excluded to enhance the reliability of the findings (Tabachnick and Fidell, 2020).

Descriptive statistics were computed to summarize demographic information (age, gender, years of experience) and the main constructs: organizational culture dimensions, supervisor support, organizational trust, and affective commitment. These statistics provided a foundational understanding of the sample and key variables.

To address our hypotheses (H1–H5) and research question (RQ1), we employed a two-step hierarchical multiple regression analysis. Continuous predictor variables were mean-centered before creating interaction terms to minimize multicollinearity (Aiken and West, 1991). In Step 1, all main effects—organizational trust, perceived supervisor support, and the four organizational culture dimensions (clan, adhocracy, market, and hierarchy)—were entered simultaneously as predictors of affective commitment. This step allowed us to assess the unique contribution of each independent variable to affective commitment while controlling for the others, thereby testing H1, H2, and H3. In Step 2, all hypothesized interaction terms were added simultaneously to the model. Specifically, this included the four interactions between perceived supervisor support and each organizational culture dimension (for H4), the interaction between perceived supervisor support and organizational trust (for H5), and the four interactions between organizational trust and each organizational culture dimension (to address RQ1).

To probe the nature of any significant moderation effects (H4, H5, and RQ1), we followed up with simple slopes analyses for all statistically significant interaction terms. This established method, as outlined by Hayes (2017), allows for a detailed examination of the conditional effects of the predictor on the outcome at low (−1 standard deviation), mean (0 standard deviation), and high (+1 standard deviation) levels of the moderator. A significance level of *α* = 0.05 was used throughout to ensure statistical robustness.

## 4 Results

### 4.1 Descriptive statistics and correlations

Table 1 provides the means, standard deviations, and intercorrelations for all study variables, explicitly detailing the four dimensions of organizational culture (clan, adhocracy, market, and hierarchy), alongside supervisor support, organizational trust, and affective commitment. All correlations were statistically significant (*p* < 0.01). Notably, strong positive relationships were observed between supervisor support and affective commitment (*r* = 0.51), as well as organizational trust and affective commitment (*r* = 0.54). The organizational culture dimensions also showed statistically significant, albeit moderate, correlations with affective commitment, ranging from *r* = 0.35 to *r* = 0.40.

**Table 1:** Descriptive statistics and correlations for study variables (*N* = 439).

Variable	M	SD	1	2	3	4	5	6	7
1. Clan (culture)	3.45	0.72	–						
2. Adhocracy (culture)	3.38	0.68	0.36**	–					
3. Market (culture)	3.29	0.74	0.34**	0.42**	–				
4. Hierarchy (culture)	3.51	0.65	0.38**	0.40**	0.39**	–			
5. Supervisor support	3.67	0.76	0.41**	0.39**	0.36**	0.38**	–		
6. Organizational trust	3.75	0.74	0.42**	0.41**	0.40**	0.43**	0.48**	–	
7. Affective commitment	3.80	0.69	0.38**	0.37**	0.35**	0.40**	0.51**	0.54**	–

**p < 0.01 (two-tailed).

Prior to conducting regression analyses, we performed a multicollinearity diagnostic on all continuous predictor variables. As some organizational culture dimensions demonstrated moderate correlations with one another (ranging from *r* = 0.34 to *r* = 0.42) and with organizational trust and supervisor support, this check was particularly important. Variance inflation factor (VIF) values for all predictors remained well below the common threshold of 5.0, and tolerance values were consistently above 0.40, confirming that multicollinearity was not a concern in this dataset.

To ensure the suitability of our data for correlation and regression analyses, we performed standard assumption checks. Normality of residuals was supported by statistically non-significant *Shapiro–Wilk* test results. Linearity between variables was visually confirmed through scatterplots, and residual plots indicated homoscedasticity, collectively supporting the appropriateness of our statistical models.

We also considered the potential for common method variance (CMV) given the single-source self-report data. To mitigate this risk, participants were assured of anonymity to reduce social desirability bias. Additionally, the survey incorporated varied scale endpoints and reverse-coded items to minimize potential for CMV. Harman’s single-factor test further suggested that CMV was not a substantial issue, as no single factor accounted for a disproportionately large portion of the variance (Podsakoff et al., 2003).

### 4.2 Hierarchical regression analysis

The hierarchical regression analysis was conducted in two steps, as detailed in Table 2. Table 2 presents the unstandardized regression coefficients (*β*), standard errors (SE), *t*-values, and *p*-values for each predictor at each step.

**Table 2:** Hierarchical regression analysis predicting affective commitment.

Predictor	Step 1 *β* (*SE*)	Step 1 *t*	Step 1 *p*	Step 2 *β* (*SE*)	Step 2 *t*	Step 2 *p*
Organizational trust	0.45 (0.04)	11.25	<0.001	0.34 (0.04)	8.50	<0.001
Supervisor support	0.23 (0.04)	5.75	<0.001	0.08 (0.06)	1.33	0.18
Clan culture	0.13 (0.05)	2.60	<0.01	0.13 (0.05)	2.60	<0.01
Adhocracy culture	0.06 (0.05)	1.20	0.23	0.06 (0.05)	1.20	0.23
Market culture	0.07 (0.05)	1.40	0.16	0.07 (0.05)	1.40	0.16
Hierarchy culture	0.11 (0.06)	1.83	<0.05	0.11 (0.06)	1.83	<0.05
Clan × supervisor support				0.03 (0.06)	0.50	0.62
Adhocracy × supervisor support				0.02 (0.06)	0.33	0.74
Market × supervisor support				0.01 (0.06)	0.17	0.87
Hierarchy × supervisor support				0.04 (0.06)	0.67	0.50
Supervisor support × organizational trust				0.23 (0.07)	3.29	<0.001
Clan × organizational trust				0.15 (0.06)	2.50	<0.05
Adhocracy × organizational trust				0.04 (0.06)	0.67	0.50
Market × organizational trust				0.03 (0.06)	0.50	0.62
Hierarchy × organizational trust				0.06 (0.06)	1.00	0.32
*R* ^2^	0.365			0.380		
*ΔR* ^2^	0.365			0.015		
*F* (Model)	63.80***			28.50***		
*ΔF*	63.80			2.05		
*p*(*ΔF*)	<0.001			<0.05		

*β* = unstandardized regression coefficient; SE, standard error. *p < 0.05, **p < 0.01, ***p < 0.001.

#### 4.2.1 Step 1: main effects (H1, H2, H3)

In Step 1, all main effect variables—organizational trust, perceived supervisor support, and the four organizational culture dimensions (clan, adhocracy, market, and hierarchy)—were entered into the model simultaneously. The model was statistically significant, *F*(6,432) = 63.80, *p* < 0.001, explaining 36.5% of the variance in affective commitment (*R*^2^ = 0.365). Organizational trust emerged as a strong positive predictor (*β* = 0.45, *SE* = 0.04, *t* = 11.25, *p* < 0.001), providing robust support for H2, while perceived supervisor support was also a statistically significant positive predictor (*β* = 0.23, *SE* = 0.04, *t* = 5.75, *p* < 0.001), supporting H3. Among the organizational culture dimensions, clan culture (*β* = 0.13, *SE* = 0.05, *t* = 2.60, *p* < 0.01) and hierarchy culture (*β* = 0.11, *SE* = 0.06, *t* = 1.83, *p* < 0.05) emerged as statistically significant positive predictors; however, Adhocracy culture (*β* = 0.06, *SE* = 0.05, *t* = 1.20, *p* = 0.23) and market culture (*β* = 0.07, *SE* = 0.05, *t* = 1.40, *p* = 0.16) did not show statistically significant effects. This provides partial support for H1.

#### 4.2.2 Step 2: interaction effects (H4, H5, RQ1)

In Step 2, the nine interaction terms were added simultaneously, including the four interactions between perceived supervisor support and each organizational culture dimension for H4, the interaction between perceived supervisor support and organizational trust for H5, and the four interactions between organizational trust and each organizational culture dimension for RQ1. This addition resulted in a statistically significant increase in variance explained (*ΔR*^2^ = 0.015, *ΔF*(9,423) = 2.05, *p* < 0.05), and the overall model remained statistically significant, *F*(15,423) = 28.50, *p* < 0.001, explaining 38.0% of the variance (*R*^2^ = 0.380). For H4, none of the interaction terms between perceived supervisor support and the organizational culture dimensions were statistically significant (clan × supervisor support: *β* = 0.03, *SE* = 0.06, *t* = 0.50, *p* = 0.62; adhocracy × supervisor support: *β* = 0.02, *SE* = 0.06, *t* = 0.33, *p* = 0.74; market × supervisor support: *β* = 0.01, *SE* = 0.06, *t* = 0.17, *p* = 0.87; hierarchy × supervisor support: *β* = 0.04, *SE* = 0.06, *t* = 0.67, *p* = 0.50), so H4 was not supported. In contrast, for H5, the interaction term perceived supervisor support × organizational trust was statistically significant (*β* = 0.23, *SE* = 0.07, *t* = 3.29, *p* < 0.001), providing strong support for H5. For RQ1, the clan × trust interaction was statistically significant (*β* = 0.15, *SE* = 0.06, *t* = 2.50, *p* < 0.05), suggesting that the positive effect of organizational trust on affective commitment is stronger in organizations with a prominent clan culture, but other interactions between organizational trust and cultural dimensions were not statistically significant (e.g., adhocracy × trust: *β* = 0.04, *SE* = 0.06, *t* = 0.67, *p* = 0.50; market × trust: *β* = 0.03, *SE* = 0.06, *t* = 0.50, *p* = 0.62; hierarchy × trust: *β* = 0.06, *SE* = 0.06, *t* = 1.00, *p* = 0.32).

### 4.3 Probing significant interactions

To further interpret the statistically significant interaction effects from Step 2, we conducted simple slopes analyses, as presented in Table 3. This analysis examined whether the effect of the predictor variable was statistically significant at low (−1 SD), mean (0 SD), and high (+1 SD) levels of the moderator.

**Table 3:** Simple slopes analysis for significant interaction effects.

Interaction	Moderator level	*β*	*SE*	*t*	*p*-value
Organizational trust × supervisor support (H5)					
	High supervisor support (+1 SD)	0.47	0.08	5.88	< 0.001
	Mean (0 SD)	0.34	0.04	8.50	< 0.001
	Low supervisor support (−1 SD)	0.25	0.09	2.78	< 0.01
Clan × organizational trust (RQ1)					
	High clan culture (+1 SD)	0.42	0.07	6.00	< 0.001
	Mean (0 SD)	0.34	0.04	8.50	< 0.001
	Low clan culture (−1 SD)	0.26	0.08	3.25	< 0.01

β = unstandardized regression coefficient; SE, standard error.

For the organizational trust × supervisor support interaction (H5), the simple slopes analysis revealed that the positive relationship between organizational trust and affective commitment was statistically significant at all three levels of supervisor support. The effect was statistically significant and positive when supervisor support was high (+1 SD: *β* = 0.47, *SE* = 0.08, *t* = 5.88, *p* < 0.001), at the mean (0 SD: *β* = 0.34, *SE* = 0.04, *t* = 8.50, *p* < 0.001), and when it was low (−1 SD: *β* = 0.25, *SE* = 0.09, *t* = 2.78, *p* < 0.01). This demonstrates that while organizational trust is a statistically significant predictor of affective commitment regardless of the level of supervisor support, its positive effect is substantially stronger when supervisor support is high.

Similarly, for the clan culture × organizational trust interaction (RQ1), simple slopes analysis revealed that the positive relationship between organizational trust and affective commitment was also statistically significant at all three levels of clan culture. The effect was statistically significant and positive when clan culture was high (+1 SD: *β* = 0.42, *SE* = 0.07, *t* = 6.00, *p* < 0.001), at the mean (0 SD: *β* = 0.34, *SE* = 0.04, *t* = 8.50, *p* < 0.001), and when it was low (−1 SD: *β* = 0.26, *SE* = 0.08, *t* = 3.25, *p* < 0.01). This indicates that a strong clan culture enhances the positive impact of organizational trust on employee commitment.

## 5 Discussion

The present study investigated the relationships between organizational culture dimensions, supervisor support, organizational trust, and affective commitment among employees in mainland China. Our findings offer significant insights into how these factors directly and interactively influence employee commitment, with important implications for both theory and practice.

### 5.1 Direct effects on affective commitment

Our analysis, which simultaneously included all main effect variables (organizational trust, perceived supervisor support, and the four organizational culture dimensions) in the first step of the regression model, revealed several key drivers of affective commitment. Organizational trust emerged as a substantial positive predictor of affective commitment, aligning consistently with prior research. This body of literature highlights employees’ belief in organizational integrity, reliability, and benevolence as a powerful driver of emotional attachment (e.g., Bastug et al., 2016; Malla and Malla, 2023; Mayer et al., 1995; Vanhala et al., 2016). When employees perceive fairness and transparency, it fosters psychological safety and predictability, which are crucial for deep emotional bonds (Frazier et al., 2015; Gustafsson et al., 2021; Hurley, 2012; Kannan-Narasimhan and Lawrence, 2012). This finding aligns with social exchange principles where trust cultivates reciprocity, leading to increased commitment (Blau, 1964; Jeong and Oh, 2017). In the distinct Chinese context, where interpersonal harmony and relational stability (*guanxi*) are highly valued, organizational trust’s importance is particularly pronounced, as it underpins a reliable work environment and reduces ambiguity (Jiang et al., 2017; Taylor et al., 2023). Our findings reinforce that organizational trust is a foundational prerequisite for cultivating a deeply committed workforce (Ugwu et al., 2014; Vanhala et al., 2016).

Perceived supervisor support also demonstrated a statistically significant positive direct effect on affective commitment within this model. This reinforces the critical role supervisors play in shaping employees’ emotional attachment (Frear et al., 2018; Ng and Sorensen, 2008; Nichols et al., 2016; Orgambídez and Almeida, 2020). Effective supervisor support extends beyond task guidance to genuine concern for well-being, resource provision, and recognition (Dawley et al., 2008; Fazio et al., 2017; Rousseau and Aubé, 2010), thereby creating a work environment where employees feel valued and secure, fostering strong emotional bonds (Li et al., 2018). This dynamic aligns with Organizational Support Theory (OST), which posits that employees perceive supervisors as organizational representatives (Eisenberger et al., 2002). Support from supervisors directly influences employees’ perceptions of overall organizational support (Rothmann and Cooper, 2015). Thus, the direct link between supervisor support and affective commitment reflects not just interpersonal ties but also reinforced perceived organizational backing (Casimir et al., 2014). When employees view supervisors as caring, this sentiment often generalizes to the organization itself, strengthening emotional attachment and willingness to remain engaged (Eisenberger et al., 2020; Rhoades and Eisenberger, 2002). Supervisor support therefore bridges direct interpersonal commitment and a deeper organizational connection.

Further examining the main effects, our results indicated that specific organizational culture dimensions differentially predicted affective commitment. Clan and hierarchy cultures were statistically significant positive predictors, while adhocracy and market cultures did not show a similar impact. These findings underscore the nuanced influence of cultural dimensions, particularly within the Chinese context, where collectivist values and respect for authority are deeply ingrained. The positive association with clan culture suggests that organizations emphasizing teamwork, participation, and a family-like atmosphere cultivate strong emotional attachments (Aranki et al., 2019; Kawiana et al., 2018). Clan cultures prioritize collaborative decision-making and open communication (Cameron and Quinn, 2006), fostering belonging and emotional security. This aligns with social exchange theory (Blau, 1964) and is crucial in collectivist cultures like China, where group harmony and relational bonds are highly valued (Hofstede, 2001; Wang, 2020). Hierarchy culture also significantly predicted affective commitment (Lin et al., 2023). While often associated with rigidity, in the Chinese context, influenced by Confucianism’s emphasis on respect for authority and social order (Hofstede, 2001), hierarchy provides stability, structure, and role clarity (Wang, 2020). This can reduce uncertainty and foster security, strengthening commitment (Schein, 2010). Consistent leadership and clear processes in hierarchical organizations provide stability and fairness, enhancing emotional connection (Ashikali and Groeneveld, 2015; Hofstede, 2001). In contrast, adhocracy and market cultures were not significant predictors. Their emphasis on innovation, risk-taking, and competition may not resonate in collectivist cultures like China, which value stability and long-term relationships over individual achievement (Hofstede, 2001). Adhocracy’s dynamism may conflict with a preference for structured environments (Wang, 2014; Jiang et al., 2017). Market culture’s results-oriented nature may prioritize external achievements over internal relational bonds, essential for affective commitment (Hofstede, 2001). These findings are consistent with research indicating that market and adhocracy cultures, focused on external adaptability, may not cultivate the same emotional engagement as clan and hierarchy cultures, which prioritize internal integration and relational bonds (Cameron and Quinn, 2006; Denison, 1990). Employees in market-oriented cultures might perceive a conflict with their need for relational support, crucial for commitment (Ashikali and Groeneveld, 2015). These results emphasize the critical role of cultural context in determining which organizational culture dimensions foster affective commitment (Chordiya et al., 2017). Our findings highlight that in China, organizational cultures aligning with collectivism, relational harmony, and respect for authority are more likely to foster affective commitment. The significant role of clan and hierarchy cultures illustrates the need for cultural alignment when enhancing employee commitment, particularly where relational ties and stability are central. This suggests that organizations operating in different cultural contexts may need tailored approaches to foster affective commitment. While entrepreneurial cultures may suit Western societies, collectivist cultures like China may benefit more from relational and stability-oriented cultures (Hofstede, 2001; Jiang et al., 2017).

### 5.2 Interaction effects on affective commitment

Beyond these direct effects, our analysis revealed crucial interactive dynamics, particularly concerning the moderating role of supervisor support and the interplay between specific cultural dimensions and trust. A key finding was the statistically significant moderating effect of supervisor support on the relationship between organizational trust and affective commitment (H5). Notably, our results indicated that supervisor support did not significantly interact with any of the organizational culture dimensions (H4 was not supported). However, the statistically significant interaction with organizational trust clearly showed that the positive association between organizational trust and affective commitment was significantly strengthened when supervisor support was high. This underscores a critical synergistic dynamic where supervisor support acts as a catalyst, maximizing organizational trust’s beneficial impact on employee commitment (Zhang et al., 2008).

Notably, our results indicated that supervisor support did not statistically significantly interact with any of the organizational culture dimensions, which means H4 was not supported. This non-significant finding is important and suggests that while supervisors serve as critical intermediaries for organizational trust, their influence in the Chinese context may not extend to moderating the broader, more deeply ingrained organizational cultural climate. It is plausible that the effects of organizational culture are more stable and less susceptible to the contingent influence of a single supervisor, who, while important, may represent a more proximal and interpersonal factor compared to the overarching cultural norms. Alternatively, the specific cultural dimensions of the competing values framework may be too broad to be effectively moderated by supervisor support. Future research could explore whether more specific, localized cultural attributes might show such a moderating effect.

Furthermore, our analysis revealed a statistically significant interaction between clan culture and organizational trust on affective commitment (RQ1). Simple slopes analysis indicated that the positive effect of organizational trust on affective commitment was stronger in organizations characterized by a high clan culture. This suggests that in environments emphasizing teamwork, collaboration, and a family-like atmosphere, the benefits of organizational trust in fostering commitment are further enhanced. This finding highlights how specific cultural values can synergize with a general climate of trust to deepen employee emotional attachment. The absence of significant interactions between other cultural dimensions (adhocracy, market, hierarchy) and organizational trust suggests that while these cultures may have direct effects on commitment, they do not consistently moderate the trust-commitment relationship in the same way that a clan-oriented culture does.

## 6 Implications

This study contributes valuable insights to the literature on organizational commitment by examining how organizational culture, supervisor support, and organizational trust collectively influence affective commitment. Our findings hold significant implications for both theory and practice.

### 6.1 Theoretical implications

The significant roles of clan and hierarchy cultures observed within the Chinese context underscore the importance of considering cultural specificity in organizational frameworks. While prior research has often generalized cultural dimensions across diverse contexts (Hofstede, 2001), these findings highlight how collectivist values, such as teamwork and respect for authority, uniquely shape affective commitment in non-Western settings (Jiang et al., 2017). For example, hierarchical structures, frequently critiqued in Western contexts for promoting rigidity, may instead foster stability and emotional attachment in cultures where structured authority is culturally valued. This emphasizes the need for context-specific research to enrich universal organizational theories.

The study also expands organizational support theory (OST) by emphasizing the centrality of supervisor support in moderating the relationship between trust and commitment. These findings highlight the role of supervisors as critical intermediaries in fostering employees’ trust in organizational systems (Eisenberger et al., 2002). By demonstrating that supervisor support amplifies the influence of organizational trust on affective commitment, this research advocates for integrating trust and support as interdependent constructs within models of organizational commitment.

Furthermore, the interaction between clan culture and trust adds nuance, showing how relational cultures can enhance trust’s effects on commitment. This study suggests the need for refining commitment frameworks to incorporate the interactive effects of trust and support, acknowledging that these dynamics are deeply embedded within specific cultural norms and values. While our study is situated within a single cultural context, it implicitly highlights that findings from Western research may not be directly generalizable to collectivist cultures like China. This makes it essential for future research to explicitly explore how localized cultural factors shape trust-support interactions and their influence on commitment outcomes across a wider range of cultural settings, thereby building truly cross-cultural commitment models.

### 6.2 Practical implications

From a practical standpoint, this study provides actionable insights for organizations seeking to strengthen affective commitment, while also offering valuable guidance for employees on how to better navigate and thrive within their professional environments. For organizations, it is crucial to consider fostering a clan culture, emphasizing teamwork, participation, and a family-like environment, which has been shown to increase employees’ emotional attachment. In the Chinese context, hierarchy culture also positively predicted affective commitment, suggesting that organizations should maintain clear structures and well-defined roles to provide the stability employees value. This is particularly important in cultures that prioritize authority and structure as sources of organizational clarity. Furthermore, understanding these dynamics provides valuable guidance for employees, helping them to identify and seek out workplaces that align with their personal needs for relational support and stability. A strong affective commitment is not just a benefit to the company; it also translates to greater job satisfaction, a stronger sense of belonging, and enhanced career resilience for the employee.

In addition, supervisor training should be a focal point for organizations. Supervisors equipped with emotional intelligence skills can better respond to employees’ emotional and practical needs, fostering a supportive environment conducive to trust and commitment. Organizations should invest in continuous leadership development programs to help supervisors create environments that enhance both organizational trust and affective commitment. Finally, maintaining high levels of organizational trust is essential. Trust-building measures such as transparency in decision-making, fairness in processes, and consistency between policies and actions should be prioritized. A trustworthy organizational environment, supported by emotionally intelligent supervisors, can significantly bolster affective commitment, leading to positive outcomes such as improved performance and reduced turnover intentions.

## 7 Limitations and future research

This study, while offering valuable insights, has certain limitations. The cross-sectional design restricts the ability to infer causality among variables, meaning that the direction of influence between organizational culture, perceived supervisor support, organizational trust, and affective commitment cannot be definitively established. Future studies should employ longitudinal designs to explore these relationships over time and assess how changes in trust, support, and culture impact affective commitment across different organizational stages. Additionally, the reliance on self-report measures introduces the possibility of common method variance (CMV). Although steps were taken to mitigate this risk, such as ensuring anonymity and conducting Harman’s single-factor test, future research could benefit from integrating objective measures or multi-source data to further minimize CMV.

Given that the study was conducted within the Chinese context, future research should explore whether the findings generalize to other cultural settings. Comparative studies across different countries could shed light on how cultural values interact with organizational dynamics to influence commitment. For instance, individualistic cultures may respond differently to the effects of clan and hierarchy cultures, and further exploration could provide a more comprehensive understanding of cultural dimensions in shaping affective commitment globally. Furthermore, while our study utilized hierarchical regression, Structural Equation Modeling (SEM) presents a valuable avenue for future research to explore these intricate relationships with a more holistic model testing approach, enabling the simultaneous assessment of direct and indirect effects within a comprehensive theoretical framework.

Another area for future research could be examining additional moderators or mediators that may influence the relationship between organizational trust and affective commitment. For example, constructs such as psychological safety, job satisfaction, or employee engagement could be explored as potential mediating factors that deepen employees’ commitment to the organization (Gao et al., 2022; Wong and Wong, 2017). This would contribute to a more nuanced understanding of the underlying mechanisms driving affective commitment, allowing organizations to design more targeted interventions.

Finally, given the critical role of perceived supervisor support in fostering commitment, future research should further explore leadership behaviors that strengthen supervisor-employee relationships. Understanding how specific supervisor actions influence employees’ trust and engagement could offer additional strategies for organizations to build a more committed and resilient workforce.

## 8 Conclusion

The study underscores the critical role of organizational culture, supervisor support, and organizational trust in fostering affective commitment among employees. Clan and hierarchy cultures were statistically significant predictors of affective commitment, highlighting the importance of cultural alignment with employee values. Supervisor support emerged as a strong predictor and a moderator that enhances the positive impact of organizational trust on affective commitment. These findings emphasize the interconnected nature of organizational factors in shaping employee commitment. Organizations aiming to enhance affective commitment should focus on cultivating supportive cultures, developing supervisors’ supportive capabilities, and building organizational trust. By addressing these areas, organizations can foster a more committed and engaged workforce, while employees can achieve greater job satisfaction, a stronger sense of belonging, and improved well-being. This ultimately contributes to improved organizational performance and reduced turnover intentions.

## Data Availability

The datasets generated and analyzed during the current study are available from the corresponding author upon reasonable request. Requests to access these datasets should be directed to Xiaoyan Zhang, nico_zxy@sina.com.
